# Melatonin treatment improves postharvest quality and regulates reactive oxygen species metabolism in “Feizixiao” litchi based on principal component analysis

**DOI:** 10.3389/fpls.2022.965345

**Published:** 2022-08-11

**Authors:** Jing Xie, Ziyi Qin, Jiali Pan, Jing Li, Xia Li, Hock Eng Khoo, Xinhong Dong

**Affiliations:** ^1^Guangxi Key Laboratory of Electrochemical and Magneto-chemical Functional Materials, College of Chemistry and Bioengineering, Guilin University of Technology, Guilin, China; ^2^South Asia Branch of National Engineering Research Center of Dairy Health for Maternal and Child Health, Guilin University of Technology, Guilin, China; ^3^Changzhou Institute of Materia Medica Co., Ltd, Changzhou, China

**Keywords:** litchi fruit, melatonin, methionine sulfoxide reductase, postharvest storage, reactive oxygen species

## Abstract

Postharvest quality of litchi reduces rapidly during storage at room temperature. This study aimed to investigate the effect of melatonin treatment on postharvest quality and oxidative stress markers of litchi fruit during cold storage. The “Feizixiao” litchi was treated with melatonin solution concentrations of 0.2 and 0.6 mmol·L^−1^ and then stored at 4°C for 12 days. The results confirmed that the melatonin treatment effectively maintained the appearance and color of the litchi fruit, suppressed the peel browning, and improved the litchi quality. The treatment also significantly enhanced the levels of endogenous melatonin, antioxidant components (total phenolics, flavonoids, and anthocyanin), and antioxidant enzyme activities of the fruit. It also inhibited the other oxidative stress markers, such as $O_{2}^{-}$ , H_2_O_2_, MDA, and protein carbonyl content, and upregulated the expressions of antioxidant and Msr-related genes. Correlation and principal component analyses further confirmed that the melatonin treatment effectively delayed the fruit senescence by enhancing the antioxidant enzyme activities and modulating peel browning and reactive oxygen species metabolism of the litchi fruit *via* regulating gene expression of the related enzymes (SOD and PPO). These findings suggested that the exogenous application of melatonin to litchi during the postharvest is an ideal way to preserve the fruit quality and delay fruit senescence.

## Introduction

Litchi (*Litchi chinensis* Sonn.) is a subtropical fruit; it is mainly cultivated in Asian countries (especially in southern China), subtropical areas of South Africa, Australia, the United States, and many other places (Sandhu et al., 2021). Litchi is a popular fruit because it has an attractive red peel, unique taste, and high nutritional value (Shafique et al., 2016; Chaikham et al., 2017; Zhang et al., 2018). The fruit is non-climacteric, harvested at the mature stage during the hot and humid summer. The increasing respiration rate during storage at ambient temperature will result in peel browning, quality deterioration, and a decline in edible quality or market value (Reichel et al., 2017). Also, water loss in the litchi pulp and peel browning are visible during the prolonged storage of the fruit. The browning of litchi peel (Guiwei variety) is attributable to the degradation of anthocyanins (Qu et al., 2021). The loss in postharvest quality is mainly due to the enzymatic and non-enzymatic reactions (Zuo et al., 2021). The enzymatic pathway involves pericarp browning caused by antioxidant enzymes. Moreover, non-enzymatic browning is related to lipid peroxidation and protein oxidation (Ventanas et al., 2007).

Melatonin, chemically known as N-acetyl-5-methoxytryptamine, is a natural indoleamine (Talib, 2018). Melatonin widely exists in living organisms. It is a multifunctional biological regulatory molecule (Sun et al., 2021b), such as growth, development, and other cycles (Lin et al., 2019). It is known to eliminate reactive oxygen radicals in plants. It also protects plants from oxidative damage by stimulating the antioxidant enzyme activity. The exogenous application of melatonin reduces cell damage and increases the activities of antioxidant enzymes by inhibiting oxidative stress (Jannatizadeh et al., 2019). The role of melatonin in the postharvest preservation of fruit has gradually become a hot topic. A review paper showed that melatonin has used in postharvest storage of several types of fruits. They are apple, banana, berries, sweet cherry, citrus, dates, grapes, kiwifruit, litchi, mango, peach, pear, plum, pomegranates, sapota, and tomato (Ze et al., 2021). Melatonin has been reported to play a crucial role in delaying postharvest fruit ripening, enhancing antioxidant activity, and a better physical appearance of apples during storage (Onik et al., 2021). Melatonin treatment could increase pomegranate fruit quality, especially the aril color and antioxidant components at harvest and storage periods (Lorente-Mento et al., 2021). The compound also improved the postharvest quality of green asparagus (Boonsiriwit et al., 2021) and mushroom (Lin et al., 2022).

Literature showed that melatonin treatment improved the postharvest quality of several varieties of litchi. The postharvest treatment reduced peel browning and regulated reactive oxygen species (ROS) metabolism, antioxidant enzyme activity, and gene expression in the Ziniangxi variety of litchi stored at room temperature of 25°C (Zhang et al., 2018). Another study reported that Baitangying variety of litchi treated with 400 μmol·L^−1^ melatonin alleviated chilling injuries and increased the antioxidant enzyme activities associated with energy metabolism (Liu et al., 2020). Another variety of litchi (*L. chinensis* Sonn. cv. “A4Wuhe”) has also been determined for its postharvest preservation effect using 400 μmol·L^−1^ melatonin (Wang et al., 2020). The melatonin treatment reduced pericarp browning by suppressing lipase, lipoxygenase, and phospholipase D activities. It also increased saturated fatty acids content. A recent paper showed that abscisic acid signaling was involved in the delayed senescence of the melatonin-treated litchi fruit (*L. chinensis* Sonn. cv. Heiye). The other genes involved in the regulation of litchi senescence were related to energy production and ROS and protein metabolisms (Zhang et al., 2021). Thus, there is a need to explore the effect of different melatonin concentrations on different litchi varieties at several storage conditions.

Several studies have reported the efficacy of melatonin in postharvest preservation of different varieties of litchi at room temperature (25°C). Limited studies have been performed to determine the cold storage effect of melatonin-treated litchi on peel browning and ROS metabolism, except for the study on prevention of chilling injury *via* regulation of energy and proline metabolisms. A systematic and comprehensive evaluation of these preservative effects is needed. Therefore, we aim to systemically investigate possible effects of exogenous melatonin treatment on postharvest quality and senescence of “Feizixiao” litchi during storage at 4°C for 12 days. Data on fruit appearance, antioxidant activities, and oxidative stress markers provide scientific evidence of efficacy of melatonin treatment on cold storage of litchi. The correlation and principal component analyses can also show the physical metabolism of melatonin-treated litchi and the fruit senescence.

## Materials and methods

### Sample preparation and treatment

Litchi (*L. chinensis* Sonn.) of the “Feizixiao” variety was harvested at commercial maturity from an orchard in Nanning city, Guangxi Zhuang Autonomous Region, China. The selected fruits were in uniform shape and weight without visual blemishes. Fruits were rinsed with distilled water and air-dried at room temperature. They were then randomly divided into three groups (300 fruits per group). The first group (control group) was soaked in distilled water; the second group was immersed with 0.2 mmol·L^−1^ melatonin solution (low-dose) for 20 min; the third group was soaked with 0.6 mmol·L^−1^ melatonin solution (high-dose) for 20 min. All fruit samples were then stored at 4°C for 12 days. The fruit sample was selected based on a random sampling of 60 fruits per group at a 3-day interval for physiological and quality evaluation. Pulp and peel of litchi were separated, frozen in liquid nitrogen, and stored at −80°C for further experiment. The doses were selected based on the findings reported in the literature (Zhang et al., 2018).

### Determination of weight loss

Weights of litchi samples were obtained using an electronic balance. The initial weights of the fruit samples were recorded as *M*_1_ (g). The masses of the fruit samples collected on 0, 3, 6, 9, and 12 days were recorded as *M*_2_ (g). The weight changes were calculated based on the equation as follows: (Osuga et al., 2021; Owolabi et al., 2021).


$$\mathrm{Weight loss}\;\left(\%\right)=\frac{M_{1}-M_{2}}{M_{1}}\times 100$$

### Determination of browning index

Browning degree of litchi was divided into five grades (Ali et al., 2018; He et al., 2020). The browning degrees were defined as follows: 1 = no browning, 2 = 0%–25% browning, 3 = 25%–50% browning, 4 = 50%–75% browning, and 5 = 75%–99% browning. The browning index of litchi peel was calculated. Each experiment was repeated three times.


$$\begin{array}{l} \mathrm{Browning index}\\ =\frac{\Sigma \left(\mathrm{Browning degree}\times \mathrm{Number of fruit}\;\mathrm{per}\;\mathrm{category}\right)}{\mathrm{Total number of fruit}\;\mathrm{per}\;\mathrm{treatment}} \end{array}$$

### Determination of peel color

The determination of litchi peel color was performed based on the CIE color system using a precision chromatometer (Shenzhen Sanenshi Technology Co., Ltd., China). It was performed according to the previous method (Zhang et al., 2018). L*, a*, b*, and c* values of litchi samples were measured. Two measurements were made at three points on each litchi. The changes in the hue of litchi were observed on 0, 3, 6, 9, and 12 days (He et al., 2019).

### Determination of superoxide anion ( $O_{2}^{-}$ ) production rate and hydrogen peroxide (H_2_O_2_) content

Superoxide anion ( $O_{2}^{-}$ ) production rates of litchi peel were measured using the hydroxylamine method (Guo et al., 2019). In brief, a 2.0 g of litchi peel powder was added to 10 mL of 50 mmol·L^−1^ phosphoric acid buffer with a pH of 7.8. The mixture was homogenized for 30 s. It was then extracted with distilled water for 10 min in an ice bath, followed by centrifuging at 4°C and 12,000 × *g* for 20 min. The supernatant (1 mL) was added with 1.0 mL of 1 mmol·L^−1^ hydroxylamine and 1.0 mL of 50 mmol·L^−1^ phosphate buffer at pH 7.8 in an empty test tube. The mixture was incubated at room temperature (25°C) for 1 h before adding 1.0 mL of 17 mmol·L^−1^ p-aminobenzene sulfonic acid and 1.0 mL of 7 mmol·L^−1^ ɑ-naphthylamine. Absorbance of the final mixture was measured after incubation at room temperature for 20 min. The O_2_^−^ production rate was calculated using sodium nitrite as standard, and the result was expressed as nmol·g^−1^·min^−1^. H_2_O_2_ content was determined using the hydrogen peroxide test kit (Nanjing Jian Cheng Institute of Biology, Nanjing, China). The H_2_O_2_ assay was performed according to the manufacturer’s instruction.

### Determination of membrane permeability

The determination of membrane permeability was performed using 30 pieces of litchi peel for each sample. The peel pieces had equal size and thickness. The assay was done following the method described in the literature with some modifications (Ali et al., 2020). The initial electrical conductivity of the peel was *X*_1_, and the total electrical conductivity after boiling was *X*_2_. The relative conductivity (*Y*) was calculated as follows:


$$Y\left(\%\right)=\frac{X_{1}}{X_{2}}\times 100$$

### Determination of malondialdehyde and protein carbonyl content

Malondialdehyde in litchi peel was analyzed using the thiobarbituric acid colorimetric method (Jedrzejuk et al., 2018; Ge et al., 2020). A 2.0 g litchi peel powder was homogenized in 10 mL of 5% trichloroacetic acid (TCA). The homogenate was centrifuged at 5000 *× g* for 20 min, and then 1 mL of supernatant was mixed with 3 mL of 0.5% thiobarbituric acid (dissolved in 10% TCA). The mixture was heated for 30 min at 100°C and cooled to room temperature in a beaker filled with ice cubes. After centrifugation at 5000 *× g* for 10 min, absorbances (*A*) of the supernatant were measured at 532 nm and 600 nm. A reagent mixture without the test sample was used as a blank. Malondialdehyde content was calculated based on an equation as follows:


$$\begin{array}{l} MDA\;\left(\mu \mathrm{mol}\cdot {\mathrm{kg}}^{-1}\right)\\ =\frac{6.45\times \left(A_{532}-A_{600}\right)-0.56\times A_{450}}{\mathrm{Sample weight}}\times \mathrm{Total volume} \end{array}$$

Protein carbonyl content of the peel samples was determined using a protein carbonyl content kit (Nanjing Jiancheng Institute of Biology, Nanjing, China). The assay was performed following the manufacturer’s instruction. Briefly, 0.1 mL of litchi peel solution (200 mg·mL^−1^) was mixed with the 0.4 mL of reagent 3. The sample mixture was homogenized and let to stand at 37°C for 30 min before adding 0.5 mL of reagent 5. It was centrifugated at 12,000 rpm for 10 min (4°C) after the addition of reagent 5. The supernatant was discarded, and the pellet was added and redissolved in 1.0 mL of ethanol-ethyl acetate mixture. The pellet was washed for another three times with ethanol-ethyl acetate mixture before adding 1.25 mL of reagent 6. The final homogenized mixture was placed in a water bath at 37°C for 15 min before final centrifugation. The control mixture was prepared by replacing reagent 3 with reagent 4. Absorbance (A) of the supernatant was measured at 370 nm. Protein carbonyl content of litchi peel was calculated according to the equation as follows:


$$\begin{array}{l} \mathrm{Protein carbonyl content}\;\left(\mathrm{nmol}\cdot {\mathrm{mg}}^{-1}\;\mathrm{protein}\right)\\ =\frac{A_{\mathrm{sample}}-A_{\mathrm{control}}}{22\times 1\;\mathrm{cm}\times \mathrm{Protein concentration of sample}\;\left(\mathrm{mg}\cdot L^{-1}\right)\;} \end{array}$$

### Determination of accumulation of endogenous melatonin and soluble protein content

The accumulation of endogenous melatonin in the litchi peel was determined using a melatonin test kit (Nanjing Jiancheng Institute of Biology, Nanjing, China). Absorbance (OD value) was measured at 450 nm using a microplate reader. The concentration of plant melatonin in the sample was calculated using a standard curve. Soluble protein of the litchi pulp was analyzed according to the method described by Gu et al. (2019). The standard curve with bovine serum protein was plotted for quantification of soluble protein content in the litchi pulp.

### Determination of non-enzymatic antioxidant compounds

Total phenolic content (TPC) of the litchi peel was determined according to the Folin–Ciocalteu method (Li et al., 2015). In brief, 200 mg·mL^−1^ of litchi peel sample was mixed with the reagents. Gallic acid was used as a standard for plotting standard curves. Total flavonoids content (TFC) was determined to use the aluminum ion-colorimetric method (Nie et al., 2020). Rutin was used as the flavonoid standard. Total anthocyanin content (TAC) was measured based on the pH differential method (Chea et al., 2019; Ji et al., 2020). The buffer solutions of pH 1.0 and pH 4.5 were prepared using potassium chloride and sodium acetate, respectively. The absorbances were measured at 520 and 700 nm for the buffer solutions of pH 1.0 and 4.5, respectively.

### Determination of oxidation and antioxidant enzyme activities

Superoxide dismutase (SOD) activity of the litchi peel was determined according to Jin et al. (2017). Catalase (CAT) activity of the litchi sample was determined according to a previous method described by Zhang et al. (2016). In brief, a 2.0 g litchi peel powder was weighed and added with 10 mL of 0.1 mmol·L^−1^ phosphate buffer of pH 7.8. The mixture was swirled for 30 s and then kept in an ice bath for 10 min. The supernatant was collected after the centrifugation at 12,000 × *g* (4°C) for 30 min. One unit (U) of SOD activity was defined as the amount of enzyme resulting in 50% inhibition of NBT reduction. The CAT activity was expressed as U·g^−1^.

Ascorbate peroxidase (APX) activity of the litchi peel was determined according to a method reported in the literature (Zhou et al., 2014). A 2.0 g litchi peel powder was weighed and added with 10 mL of 0.1 mmol·L^−1^ potassium phosphate buffer (pH 7.5). The mixture was vortexed for 30 s, followed by standing in an ice bath for 10 min, and centrifugated at 12,000 × *g* (4°C) for 30 min. The supernatant was collected to determine APX activity, and the absorbance was measured at 290 nm. The APX activity was expressed as U·g^−1^.

Glutathione reductase (GR) activity of the litchi peel was determined based on the method described by Hu et al. (2020). In brief, a 2.0 g of litchi peel powder was weighed and added with 10 mL of 0.1 mmol·L^−1^ sodium phosphate buffer (pH 7.5). The mixture was homogenized for 20 s, and the enzyme was extracted in an ice bath for 10 min. The supernatant was collected after the centrifugation. The absorbance of the supernatant was measured. The GR activity was expressed as U·g^−1^.

Peroxidase (POD) activity of the litchi peel was determined according to the method described by Elsherbiny et al. (2021). Briefly, the peel sample (2.0 g) was weighed and added to the 10 mL of 0.1 mmol·L^−1^ phosphoric acid buffer solution (pH 6.0). The absorbance of the supernatant was measured at 420 nm. The POD activity was expressed as U·g^−1^ sample. Polyphenol oxidase (PPO) activity was determined according to the established method (Xu et al., 2020). The peel sample (2 g) was weighed and dissolved in 10 mL of 0.1 mmol·L^−1^ acetic acid–sodium acetate buffer solution of pH 5.5, swirled for 20 s and kept in an ice bathtub for 10 min. The mixture was centrifuged at 4°C for 30 min at 12,000 × *g*. The supernatant was collected, and the absorbance was measured.

### Gene express analysis

The relative expressions of antioxidant and methionine sulfoxide reductase (Msr)-related genes were determined using quantitative reverse transcription (qRT)-PCR. Total RNA was extracted using a rapid RNA extraction kit (Cross Ocean Biotechnology Co., Ltd., Beijing, China) following the manufacturer’s instruction. The extracted RNA was used in cDNA synthesis by a reverse transcription PCR system. For qRT-PCR, SYBR Green PCR Master Mix (CWBio, Jiangsu, China) was used for the reaction. SYBR Green Master fluorescent dye was used to analyze the relative expression levels of the genes. The relative expression was calculated by the 2^−ΔΔCT^ Ct method (Yan et al., 2020; Li et al., 2021). All primers of the qRT-PCR are listed in Table 1. The relative expression levels of antioxidant enzymes were determined using the litchi housekeeping gene Actin as endogenous control (Zhong et al., 2011). Litchi steward gene LcGAPDH was used as an endogenous control gene with the Msr family gene (Zhang et al., 2018).

**Table 1:** Names and serial numbers of litchi amplified primers (5′–3′).

Genes	Serial number	Primer sequence (5′–3′)
*Antioxidant-related genes*		
PPO	JF926153.1	GGAGCAAGAAGGAGAAGGAA GGAACCAAAGTTACCACCAC
Fe-SOD	JN671967.2	GATTGGAAGAGACAAACAC TCTACATACCCACGATGAT
Actin (reference gene)	HQ588865.1	ACCGTATGAGCAAGGAAATCACTG TCGTCGTACTCACCCTTTGAAATC
*Msr-related genes*		
LcMsrA1	KY475577.1	AGCGGTGGGTGTAGTCA CGAGCCTGGTCTTCATT
LcMsrA2	KY475578.1	AGGCTTTATGCACAGTCCC GCGTTCCCACATCATTACC
LcMsrB1	KY475579.1	GTTATCAAATCGGAGGAGG GAACTTGGCATCAGACTTGTA
LcMsrB2	MH396620	CATCATTTCATCAACCCAT AGCATCACATCCAGCACA
LcGAPDH (reference gene)	JF759907.2	GGATTTGGAAGGATTGG GGACCGTGAACACTGTCGTA

### Principal component analysis (PCA) and comprehensive evaluation

The effect of melatonin treatment on the storage quality of litchi fruits was further explored using the principal component coefficient. It featured the vector value and data standardization (Table 2). A comprehensive evaluation model was constructed using the calculated scores to show the effect of melatonin treatment on the freshness of postharvest storage of litchi. The equations used to calculate these scores are as follows:


$$\begin{array}{l} F1=0.07X_{1}+0.072X_{2}-0.058X_{3}-0.068X_{4}-0.062X_{5}\\ -\;0.068X_{6}+0.07X_{7}+0.071X_{8}+0.071X_{9}\\ +\;0.07X_{10}+0.07X_{11}+0.012X_{12}-0.032X_{13}\\ -\;0.066X_{14}-0.068X_{15}+0.016X_{16}-0.013X_{17}\\ -0.019X_{18}-\;0.029X_{19}-0.033X_{20}+0.009X_{21}\\ +0.037X_{22}-0.056X_{23}+\;0.023X_{24}+0.047X_{25}\\ +0.029X_{26}+0.033X_{27}+0.001X_{28} \end{array}$$

$$\begin{array}{l} F2=0.035X_{1}+0.001X_{2}-0.066X_{3}-0.007X_{4}\\ \;+0.026X_{5}+0.001X_{6}-0.021X_{7}-0.006X_{8}\\ \;+0.016X_{9}-0.016X_{10}+0.006X_{11}\\ \;+0.124X_{12}+0.115X_{13}-0.027X_{14}\\ \;+0.025X_{15}+0.056X_{16}+0.118X_{17}\\ \;+0.121X_{18}+0.112X_{19}+0.112X_{20}\\ \;+0.127X_{21}+0.072X_{22}-0.074X_{23}\\ \;+0.07X_{24}-0.017X_{25}-0.001X_{26}\\ \;-0.021X_{27}+0.005X_{28} \end{array}$$

**Table 2:** Loading coefficients and eigenvectors of the principal components.

Items	Loading coefficients		Eigenvectors	
	Principal component 1	Principal component 2	Principal component 1	Principal component 2
Weight loss rate	0.953	0.257	0.070	0.035
Browning index	0.982	0.001	0.072	0
L* value	−0.792	−0.493	−0.058	−0.066
a* value	−0.921	−0.054	−0.068	−0.007
b* value	−0.847	0.192	−0.062	0.026
c* value	−0.930	0.078	−0.068	0.01
H_2_O_2_	0.971	−0.047	0.070	−0.021
$O_{2}^{-}$	0.957	−0.154	0.071	−0.006
Cell membrane permeability	0.970	0.118	0.071	0.016
MDA	0.956	−0.121	0.070	−0.016
Protein carbonyl content	0.953	0.041	0.070	0.006
TPC	0.158	0.922	0.012	0.124
TFC	−0.434	0.855	−0.032	0.115
TAC	−0.894	−0.204	−0.066	−0.027
Soluble protein content	−0.922	0.182	−0.068	0.025
Melatonin content	0.221	0.415	0.016	0.056
SOD activity	−0.178	0.879	−0.013	0.118
CAT activity	−0.252	0.901	−0.019	0.121
APX activity	−0.396	0.835	−0.029	0.112
GR activity	−0.450	0.828	−0.033	0.112
POD activity	0.124	0.945	0.009	0.127
PPO activity	0.504	0.537	0.037	0.072
Fe-SOD gene expression	−0.757	−0.548	−0.056	−0.074
PPO gene expression	0.312	0.834	0.023	0.112
LcMsrA1 gene expression	0.639	−0.125	0.047	−0.017
LcMsrA2 gene expression	0.396	−0.006	0.029	−0.001
LcMsrB1 gene expression	0.451	−0.154	0.033	−0.021
LcMsrB2 gene expression	−0.003	−0.041	0	−0.005

The equation for calculating the comprehensive score is *F* = 0.48.66*F*1 + 0.2651*F*2, where *F*1 and *F*2 are respectively related to the first and second principal components, and *X_i_* (*X*_1_ − *X*_28_) is the standard data.

### Statistical analysis

All data were expressed as mean ± standard error of the mean of three replicates. Data were processed in Excel, and the graphs were drawn by Origin 9.0. The mean differences between groups were statistically analyzed based on the analysis of variance coupled with the *post hoc* LSD test. The correlation and PCA were performed to use SPSS version 23.0.

## Results

### Changes in fruit appearance, browning index, weight loss rate, and chroma

**Figure 1:**
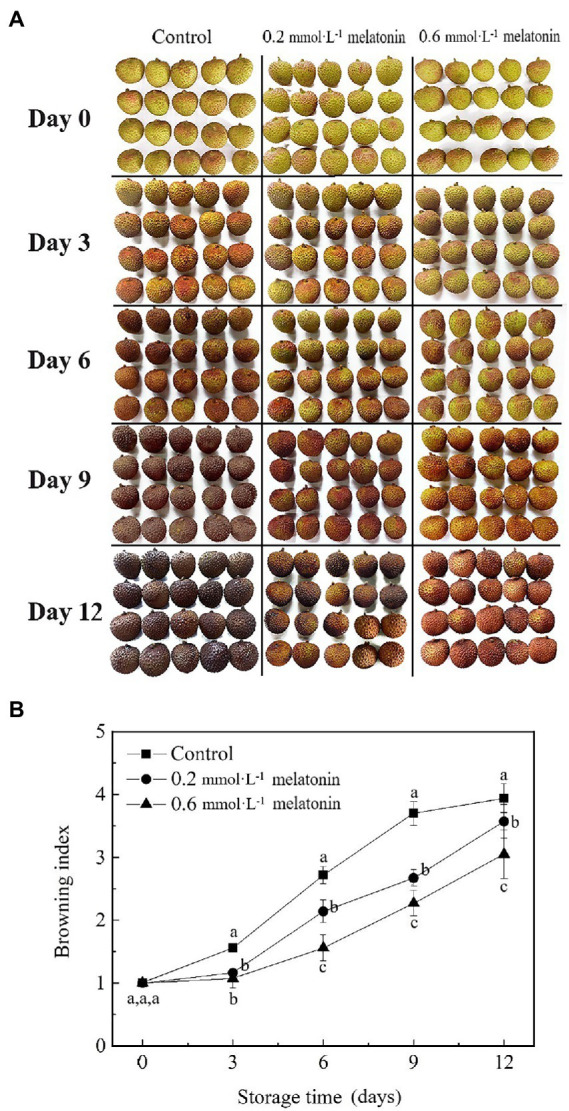
**(A)** Visual appearance and **(B)** browning index of the litchi samples during cold storage. Data represent mean values and standard errors of three replicates. Different lowercase letters (a–c) indicate significant differences between different groups (*p* < 0.05).

**Figure 2:**
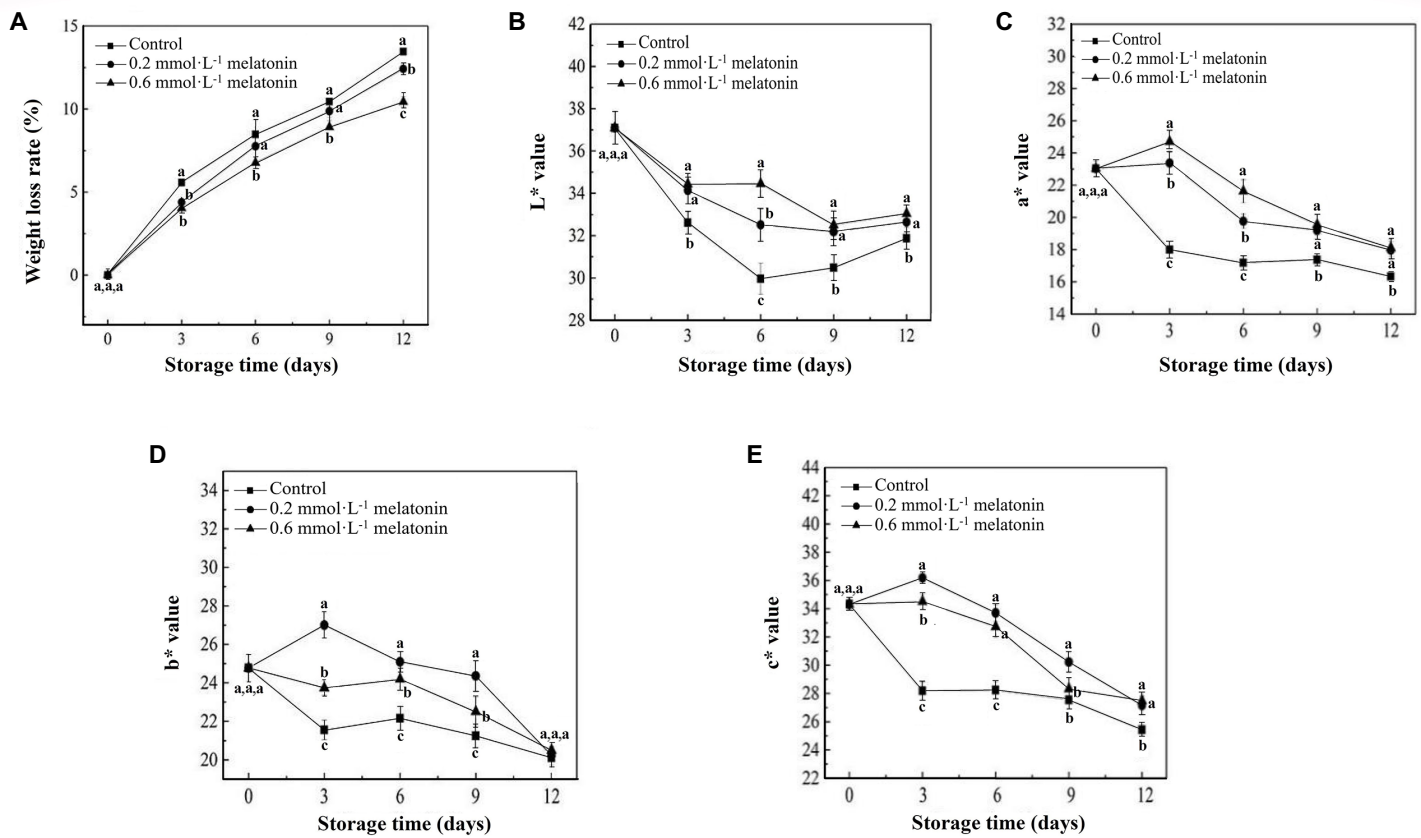
**(A)** Weight loss rate, **(B)** L*, **(C)** a*, **(D)** b*, and **(E)** c* values of the peel samples during cold storage. Data represent mean values and standard errors of three replicates. Different lowercase letters (a–c) indicate significant differences between different groups (*p* < 0.05).

The appearance change is an index that reflects the physiological metabolism of litchi, which will determine the edible quality and commercial value. Compared with the control group, the appearance of melatonin-treated litchi was much brighter, and the melatonin treatment exhibited a positive effect on intrinsic red-green color retention (Figure 1A). The prolonged storage of litchi caused a continuous increase in the browning index (Figure 1B). However, the increased rate of the browning index of melatonin-treated litchi was significantly lower than that of the control, which was consistent with the appearance retention (Figures 1A,B). The browning index of the 0.6 mmol·L^−1^ melatonin-treated fruit was significantly lower than that of the 0.2 mmol·L^−1^ melatonin treatment and the control after 3 days of storage (*p* < 0.05).
Litchi is highly susceptible to water loss due to its thin peel. The weight-loss rates of litchi fruit showed an increasing trend during the cold storage (Figure 2A). At the end of the storage period (12 days), the weight-loss rates of the litchi treated with 0.2 and 0.6 mmol·L^−1^ melatonin were 7.67% and 22.46% lower than the control, respectively.
L* value represents the fruit brightness, a* value represents the degrees of red hue (positive value) or green hue (negative value) of the fruit, b* value represents the degrees of blue hue (positive value) and yellow hue (negative value) of the fruit, and c* value represents the fruit color saturation. As the storage time increased, the lightness and chroma of the litchi peel gradually decreased. The L*, a*, b*, and c* values of the control group were significantly lower than that of the melatonin-treated group (*p* < 0.05; Figures 2B−E). Therefore, melatonin treatment can effectively maintain the appearance and chroma, reduce water loss and peel browning.

### Reactive oxygen metabolism in litchi samples

**Figure 3:**
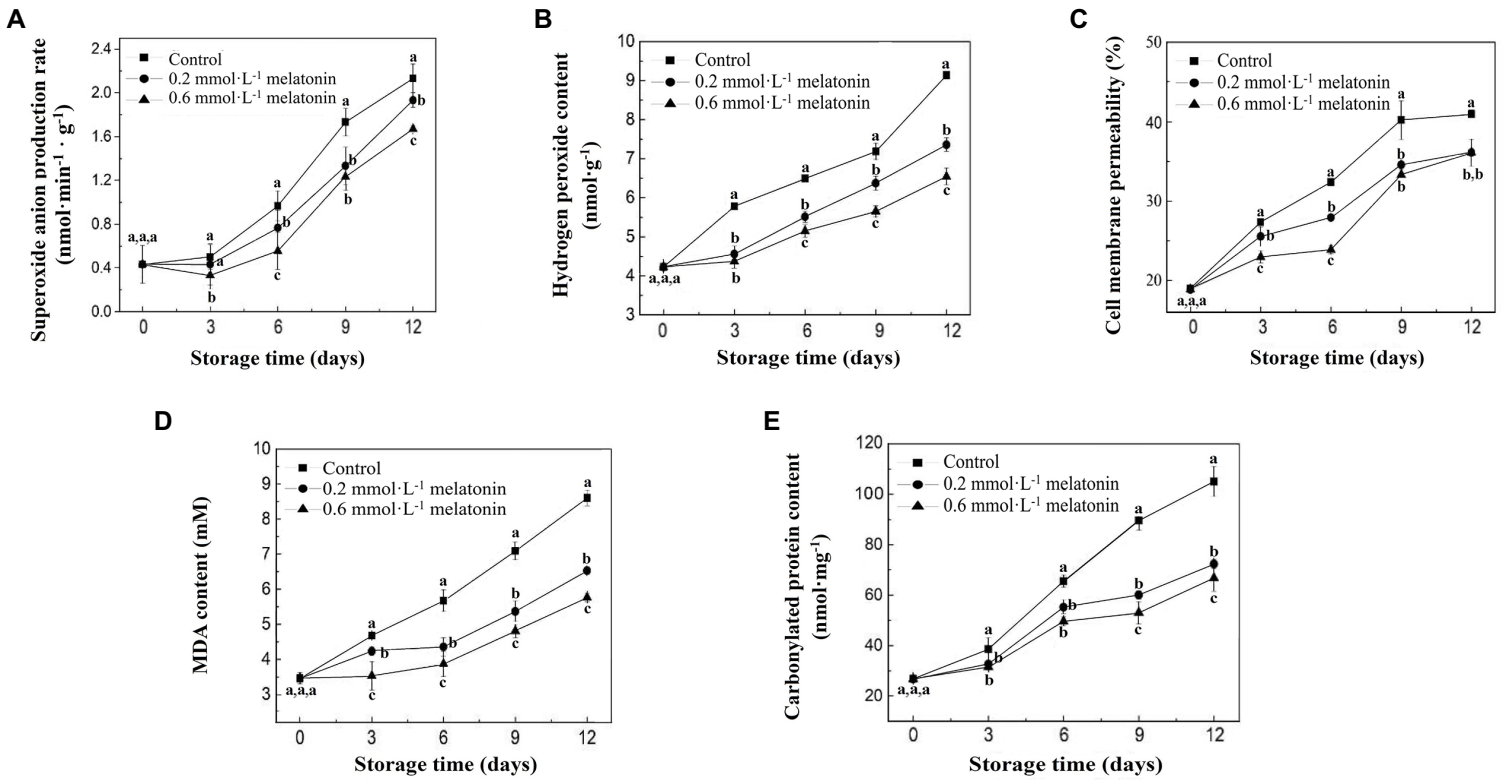
**(A)** $O_{2}^{-}$ production rate, **(B)** H_2_O_2_ content, **(C)** cell membrane permeability, **(D)** MDA, and **(E)** protein carbonyl content of the peel samples during cold storage. Data represent mean values and standard errors of three replicates. Different lowercase letters (a–c) indicate significant differences between different groups (*p* < 0.05).

During the storage of litchi samples at 4°C, the $O_{2}^{-}$ production rate and H_2_O_2_ content of litchi fruit showed a gradual increment (Figure 3A,B). Both ROS levels of the melatonin-treated groups were significantly lower than that of the control (*p* < 0.05), which suggested that melatonin effectively suppressed the accumulation of ROS. The cell membrane permeability of fruits in each group showed an increasing trend (Figure 3C). The control group had a significantly higher cell membrane permeability than both melatonin treatment groups (*p* < 0.05). At the end of storage, the cell membrane permeability values for the control, 0.2, and 0.6 mmol·L^−1^ melatonin treatment samples were 40.98%, 36.11%, and 36.09%, respectively.

Malondialdehyde and protein carbonyls are the products of lipid oxidation and protein oxidation, respectively. During storage at 4°C, MDA and protein carbonyl content of all litchi fruit showed an increasing trend (Figures 3D,E). At the end of cold storage, the MDA content of 0.2 and 0.6 mmol·L^−1^ melatonin-treated groups were 24.10% and 33.01% lower than that of the control group (8.60 mmol·L^−1^), respectively. The protein carbonyl content of 0.2 and 0.6 mmol·L^−1^ melatonin-treated groups were 31.11% and 33.01% lesser than the control group (105 nmol·mg^−1^), respectively. The results indicated that melatonin treatment reduced the accumulation of ROS under low-temperature storage and effectively inhibited the macromolecular oxidation of litchi.

### Non-enzymatic antioxidant compounds

**Figure 4:**
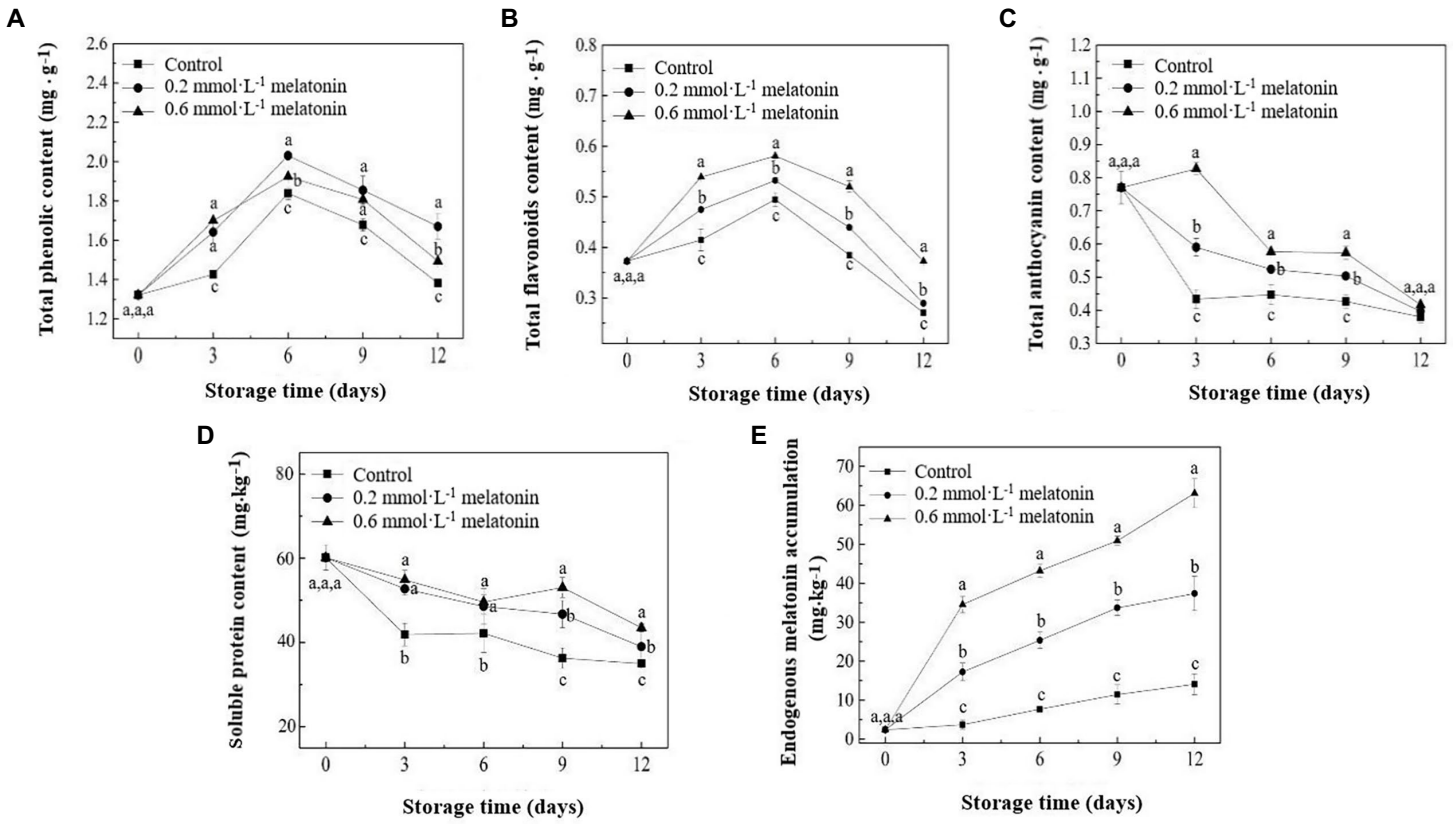
**(A)** Total phenolic content, **(B)** total flavonoids content, **(C)** total anthocyanin content, **(D)** soluble protein content, and **(E)** endogenous melatonin accumulation of the litchi samples during cold storage. Data represent mean values and standard errors of three replicates. Different lowercase letters (a–c) indicate significant differences between different groups (*p* < 0.05).

The results showed that TPC and TFC of litchi samples increased from day 0 to day 6 and then decreased from day 6 to day 12 (Figures 4A,B). Highest TPC of control, 0.2, and 0.6 mmol·L^−1^ melatonin-treated samples were determined on day 6, where the TPC values were 1.84, 2.03, and 1.92 mg·g^−1^, respectively. The highest TFC of the control, 0.2 and 0.6 mmol·L^−1^ melatonin-treated group were 0.49, 0.53, and 0.58 mg·g^−1^, respectively. The TAC of the litchi samples showed a reducing trend during storage (Figure 4C). Melatonin-treated samples had higher TAC than the control sample. The change in the TAC trend was consistent with the change in appearance, browning incidence, and color (Figures 1, 2).

The soluble protein content of the litchi pulp samples showed a reducing trend during the cold storage (Figure 4D). However, both melatonin-treated pulp samples had higher soluble protein content than the control sample. Endogenous melatonin accumulation in the peel of 0.2 and 0.6 mmol·L^−1^ melatonin-treated litchi was significantly higher than that of the control fruit peel (Figure 4E). On day 12 of the cold storage, the endogenous melatonin content of control, 0.2, and 0.6 mmol·L^−1^ melatonin-treated samples were the highest, where the values were 14.07, 37.36, and 63.10 mg·kg^−1^ sample, respectively. The results indicated that exogenous melatonin treatment strongly induced or stimulated the generation and accumulation of endogenous melatonin in the fruit.

### Antioxidant enzymes and browning-related enzyme activities

**Figure 5:**
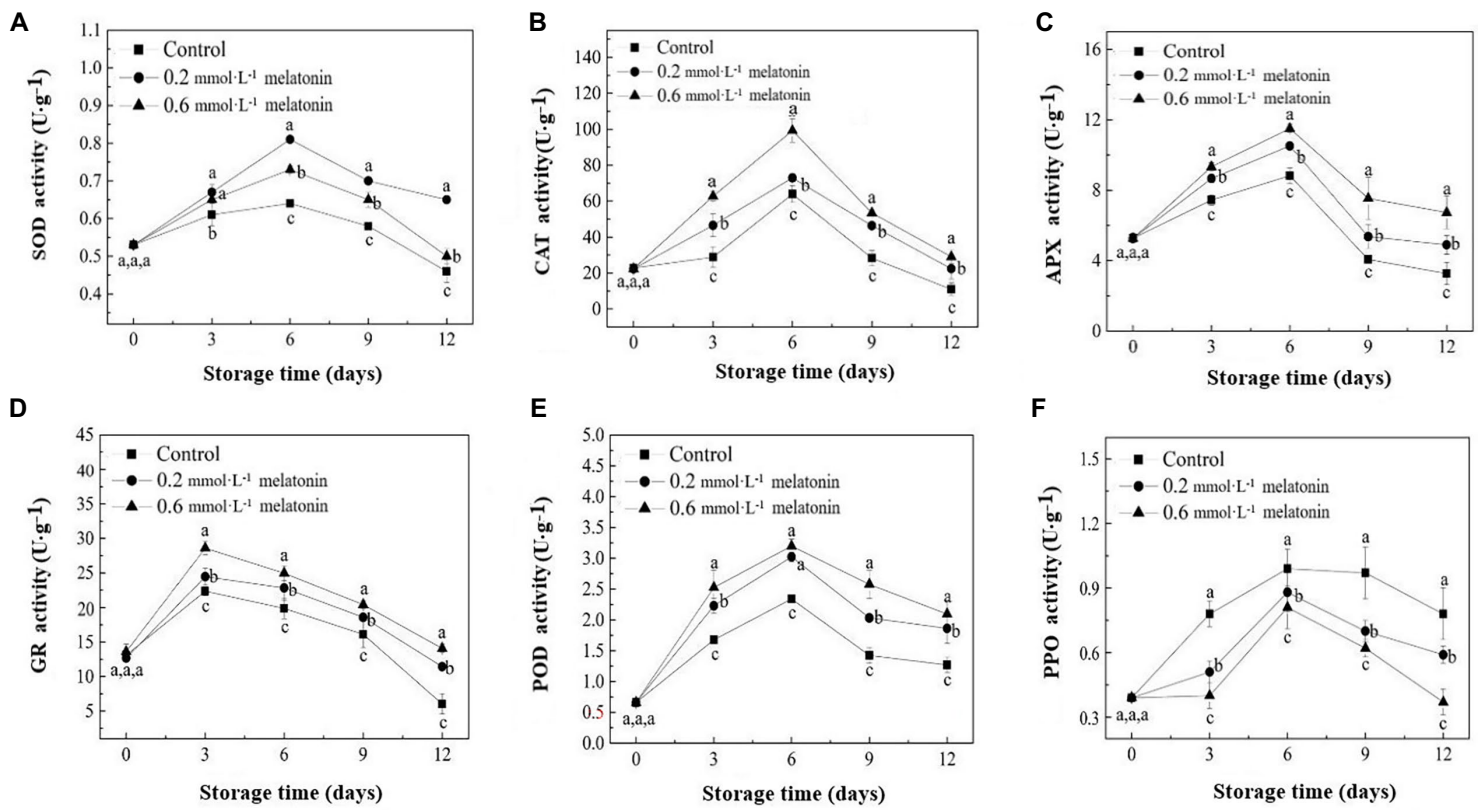
**(A)** SOD, **(B)** CAT, **(C)** APX, **(D)** GR, **(E)** PPO, and **(F)** POD activities of the peel samples during cold storage. Data represent mean values and standard errors of three replicates. Different lowercase letters (a−c) indicate significant differences between different groups (*p* < 0.05).

Superoxide dismutase, CAT, and APX activities were initially increased from day 0 to day 6 and then decreased on day 6 until the end of the storage (Figures 5A−C). On day 6, SOD, CAT, and APX activities of all groups reached a maximum level. The SOD activities of the control, 0.2, and 0.6 mmol·L^−1^ melatonin-treated samples on day 6 were 0.64, 0.81, and 0.73 U·g^−1^, respectively; the CAT activities were 63.87, 72.93, and 99.23 U·g^−1^, respectively; the APX activities were 8.83, 10.51, and 11.50 U·g^−1^, respectively. GR activity of the litchi fruits had a downward trend (Figure 5D). At the end of the storage (day 12), the GR activities of the 0.2 and 0.6 mmol·L^−1^ melatonin-treated group were 89.55% and 133.33% higher than the control group (6.03 U·g^−1^), respectively.

Peroxidase and PPO activities of the litchi fruit are shown in Figures 5E,F. The results showed that the enzymatic activities of both enzymes had a downward trend, and the enzyme activities reached maximum values on day 6. The POD activities of the control, 0.2, and 0.6 mmol·L^−1^ melatonin-treated samples were 2.34, 3.02, and 3.2 U·g^−1^, respectively. At the end of the storage, the POD activities of 0.2 and 0.6 mmol·L^−1^ melatonin-treatment group were 46.72% and 65.12% higher than that of the control group (1.27 U·g^−1^), respectively. Therefore, melatonin treatment could effectively increase SOD, CAT, APX, GR, and POD activities. Hence, the compound suppressed PPO activity of the treated litchi samples.

### Reactive oxygen metabolism-related enzyme gene expressions

**Figure 6:**
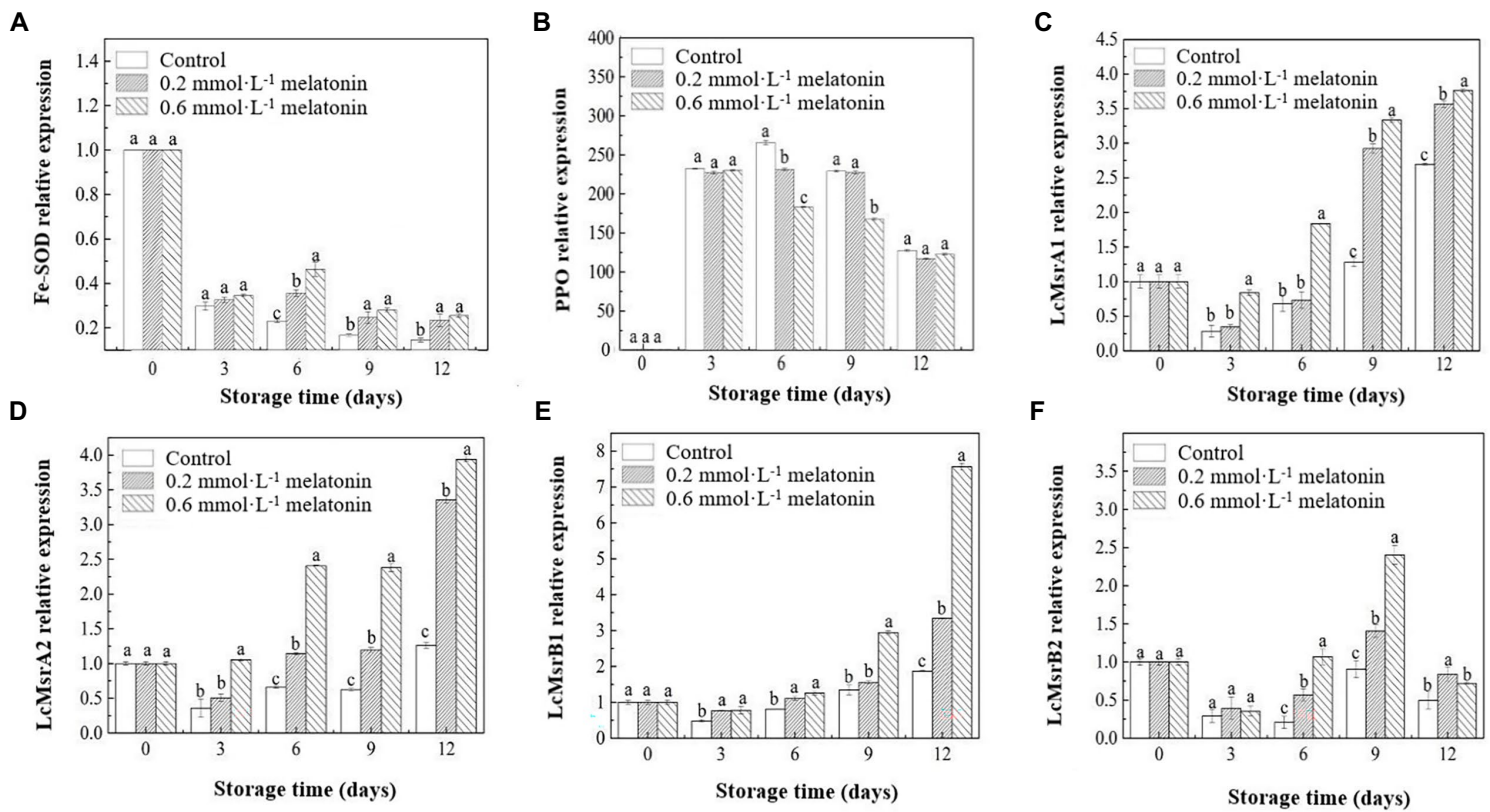
Relative expression levels of **(A)** Fe-SOD, **(B)** PPO, **(C)** LcMsrA1, **(D)** LcMsrA2, **(E)** LcMsrB1, and **(F)** LcMsrB2 of the peel samples during cold storage. Data represent mean values and standard errors of three replicates. Different lowercase letters (a−c) indicate significant differences between different groups (*p* < 0.05).

The relative expressions of the Fe-SOD gene decreased during the cold storage. The melatonin treatments upregulated the relative expression of the Fe-SOD gene compared with the control group (Figure 6A). The relative expression of the PPO gene showed a downward trend (Figure 6B). On day 6, the PPO gene of the control group was significantly upregulated (*p* < 0.05). But the gene expressions were significantly lower in the treatment groups, especially in the 0.6 mmol·L^−1^ melatonin-treated sample. The PPO gene expressions of all litchi fruits were downregulated after day 6 of the storage. Hence, the treatment failed to suppress the PPO gene expression on day 12 of the storage. As shown in Figure 1, the browning of the litchi peel is remarkable. Therefore, melatonin treatment could maintain the freshness of litchi for lesser than 12 days. The results of PPO activities also revealed that the melatonin-treated litchi samples had lower PPO activities than the control sample.

During the low-temperature storage, the relative expressions of the Msr-related genes in the melatonin-treated and control fruit were downregulated on day 3 and gradually upregulated after day 3 until the end of storage. The litchi methionine sulfoxide reductases (LcMsr)A1, LcMsrA2, LcMsrB1, and LcMsrB2 genes in the litchi samples were upregulated since the third day of storage (Figures 6C−F). At the end of the cold storage (day 12), the relative expression of the LcMsrA1 gene in the 0.2 and 0.6 mmol·L^−1^ melatonin-treated samples were 0.32-fold and 0.39-fold higher than the control sample, respectively (Figure 6C); the relative expression of LcMsrA2 gene in the 0.2 and 0.6 mmol·L^−1^ melatonin-treated samples increased by 1.67-fold and 2.13-fold (Figure 6D) and the relative expression of the LcMsrB1 gene escalated by 0.79-fold and 3.04-fold (Figure 6E), respectively. The upregulation of the LcMsrB1 gene in 0.6 mmol·L^−1^ melatonin-treated fruit was the highest on day 12 of the cold storage. Also, the relative expression of the LcMsrB2 gene increased by 0.40-fold and 1.67-fold on day 9, respectively, and the relative expression of this gene in 0.6 mmol·L^−1^ melatonin-treated sample was significantly upregulated. It was downregulated on day 12 (Figure 6F). Moreover, the 0.6 mmol·L^−1^ melatonin-treated sample had a significantly higher relative expression of these Msr-related genes than the control samples (*p* < 0.05), except for LcMsrB2. The results indicated that 0.6 mmol·L^−1^ melatonin effectively upregulated the relative expression of Msr-related genes. Thus, it contributed to the repair of the damaged protein structure.

### Correlation analysis

**Figure 7:**
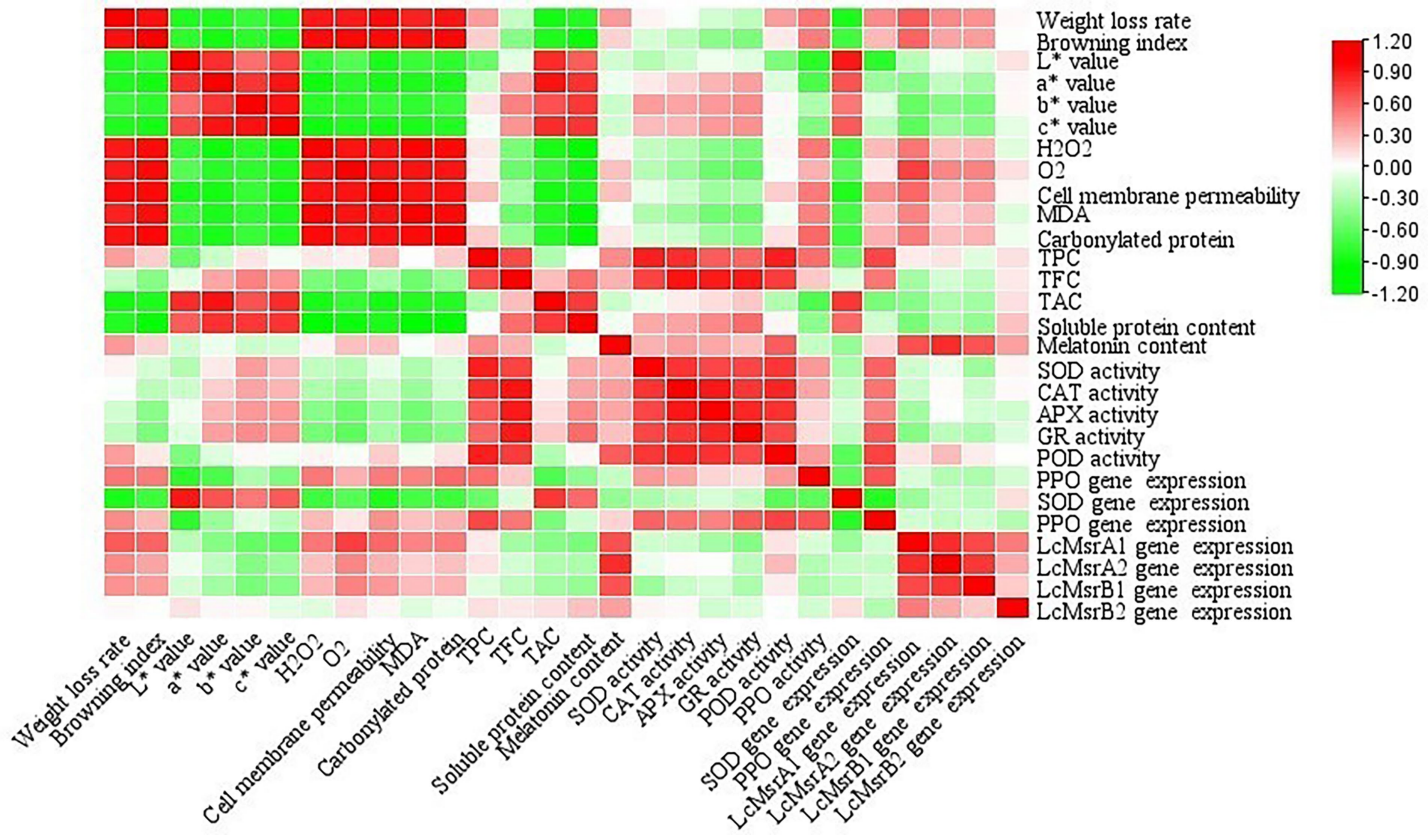
Correlation coefficients of the selected biochemical parameters determined in the litchi samples during cold storage. Different color scales indicate the different correlation coefficients.

The correlation coefficient values between all the physiological parameters are shown in Figure 7. The results showed that the browning index, cell membrane permeability, $O_{2}^{-}$ , H_2_O_2_, MDA, and protein carbonyl content were positively correlated with weight loss rate. Negative correlations were found between melatonin content and chromaticity indexes, melatonin content, and soluble protein content. Positive correlations were found between the chromaticity indexes. On the contrary, negative correlations were found between ROS and antioxidant enzyme activities.

### Principal component analysis

**Figure 8:**
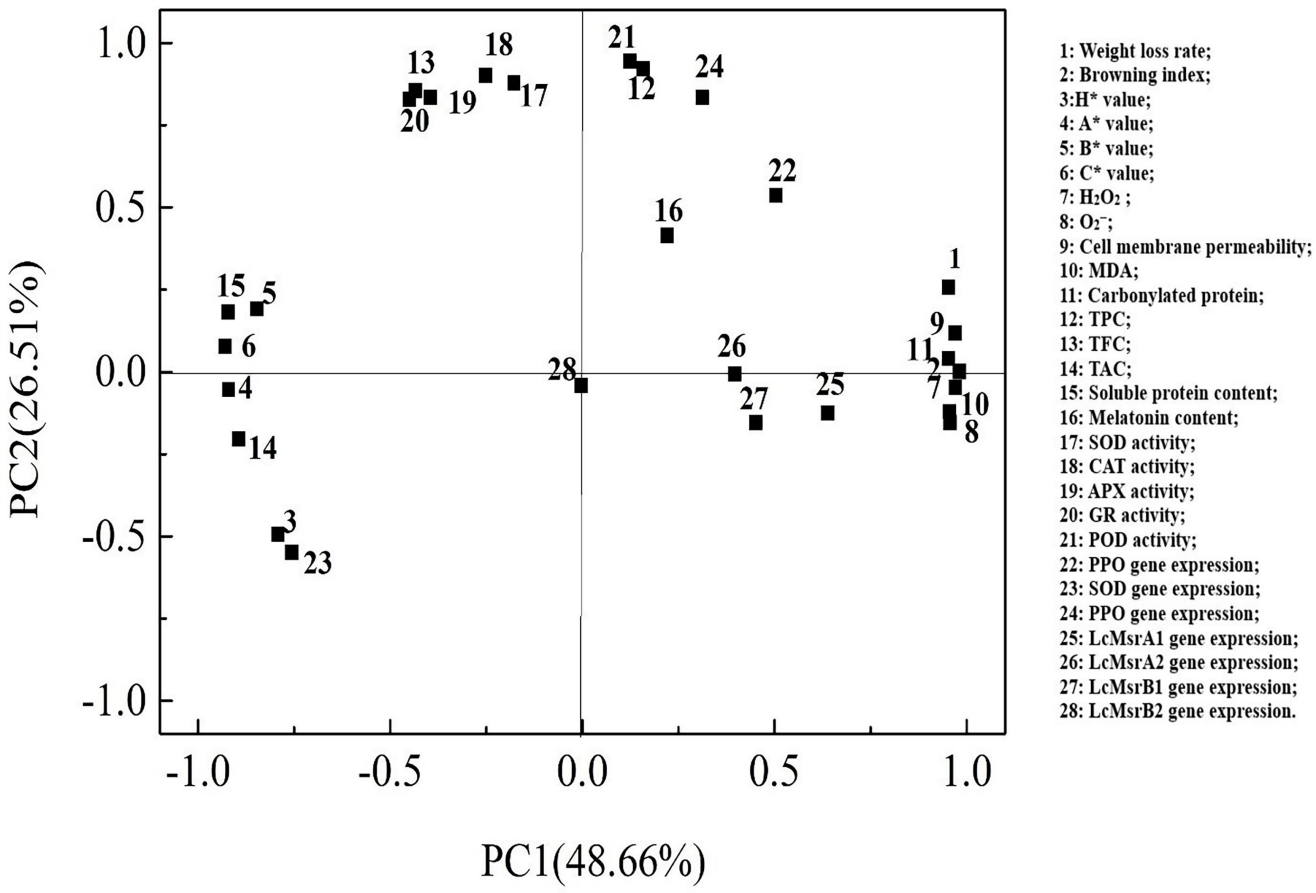
Factor loading diagram of physiological indexes of the control, 0.2, and 0.6 mmol·L^-1^ melatonin-treated samples on 0, 3, 6, 9, and 12 days of the cold storage.

**Figure 9:**
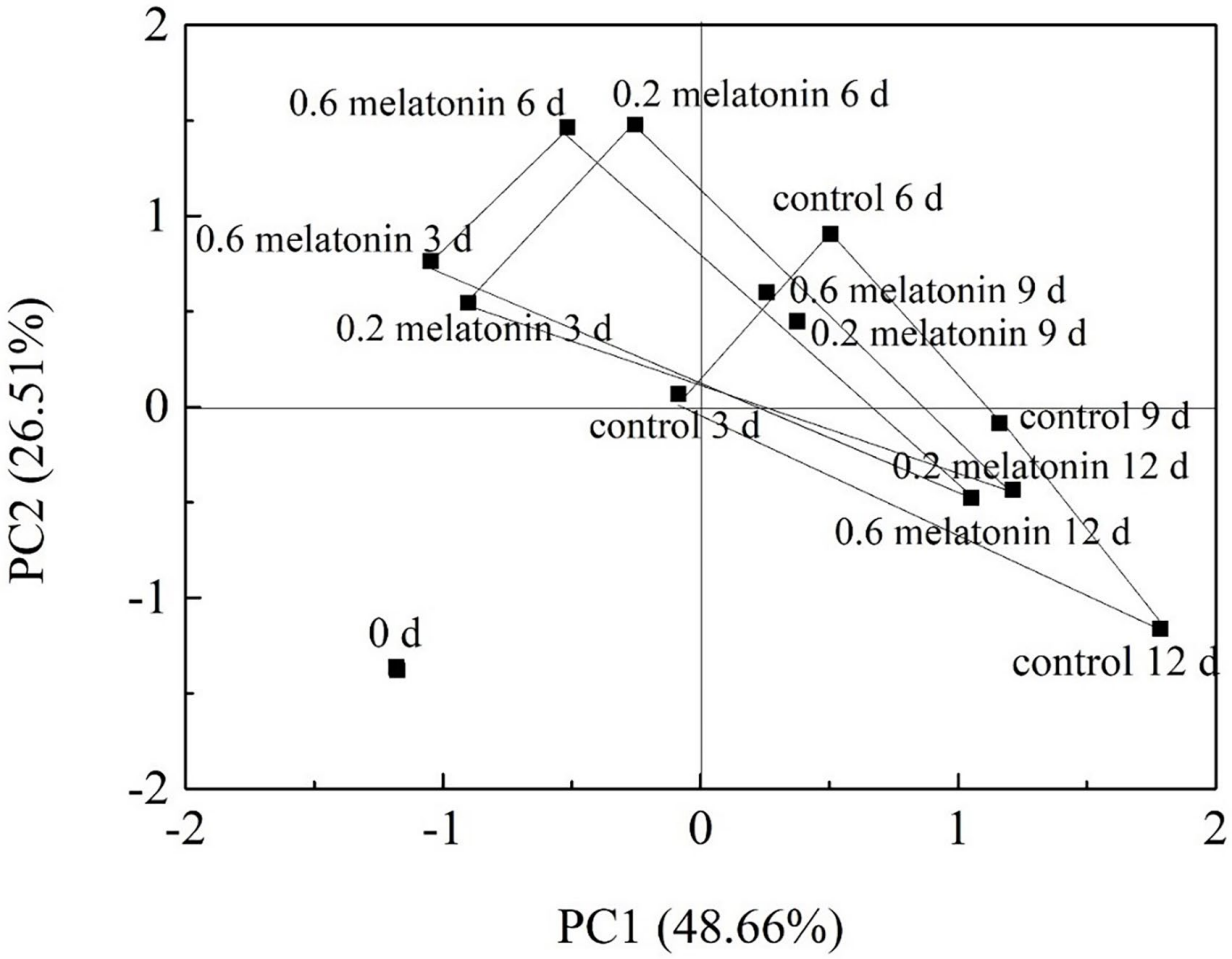
Principal component score plots of the control, 0.2, and 0.6 mmol·L^-1^ melatonin-treated samples on 0, 3, 6, 9, and 12 days of the cold storage.

Loading values and scores in the PCA enabled visualization of similarities and differences in physicochemical parameters of the studied litchi samples. PCA allowed discrimination among the different treatments (Figures 8, 9). The two components accounted for 79.80% of the variation in the dataset. The most influential parameters on the first principal component 1 (PC1) accounted for 48.66% of the variance (Figure 8). These parameters were browning index, H_2_O_2_, and cell membrane permeability. The main contributions to the second principal component 2 (PC2), which accounted for 26.51% of the variance, were POD and CAT activities.

The cumulative percent variance (CPV) of the two principal variables was 75.17% of the total variance, which met the general requirement for CPV, where the value should be >70 (Chen et al., 2021). The resulting data were presented as a representative PCA score plot of the two principal components (Figure 9). The data distribution of the melatonin-treated samples from day 3 to day 12 of the cold storage has distant from the control sample. The results suggested that the physicochemical properties of the melatonin-treated litchi samples were better than the control sample. On the PC1 axis, the melatonin-treated fruit samples could be better separated from the control sample. These results showed that PC1 could distinguish the effect of melatonin treatment on the freshness of litchi fruit.

### Comprehensive score of PCA

Principal component analysis results showed that the curves of comprehensive score plots have a downward trend (Supplementary Figure S1). The comprehensive score of the control group reduced since day 1 of the storage, whereas the scores of the melatonin-treated litchi samples increased to their maximum values on day 3. The scores began to drop drastically from day 6 to day 12. It indicated that the quality of litchi gradually reduced after day 6 of the low-temperature storage.

The comprehensive scores of the litchi samples treated with the two doses of melatonin were significantly higher than the control sample (*p* < 0.05). The comprehensive score of the 0.6 mmol·L^−1^ melatonin-treated litchi sample was higher than the score of the 0.2 mmol·L^−1^ melatonin-treated fruit sample, but no significant difference in the score was found between these two samples. Therefore, the melatonin treatment could maintain the litchi quality. The 0.6 mmol·L^−1^ melatonin was the ideal dose for use in the postharvest preservation of litchi.

## Discussion

This study determined the effect of melatonin treatment on litchi quality and its mechanism, where the fruit samples were stored at 4°C for 12 days. The application of melatonin in the postharvest preservation of litchi during low-temperature storage showed a reduced weight loss rate, inhibited browning index, and no significant changes in the litchi peel color. Therefore, it effectively maintained the cell structure and prevented rapid water loss. The browning of the litchi peel was also evaluated. The antioxidant metabolism in the fruit sample was also determined. Moreover, the melatonin treatment effectively regulated the SOD, CAT, APX, GR, and POD activities in the litchi samples (Figure 4). The enzymes play essential roles in the scavenging of ROS activity.

Literature shows that water loss is one of the factors of mango senescence and fruit shrinking (Xiao et al., 2021a). Liu et al. (2021b) reported that oxidative damage led to the destruction of cell walls of blueberry fruits, resulting in the loss of moisture. Xiao et al. (2021b) also showed that the melatonin treatment for apple and pear reduced the browning index of the fruits, increased the L* value, and effectively maintained the fruit appearance. The finding is supported by the data reported in a previous study that the melatonin treatment significantly improved the color of grapefruit (Xia et al., 2021).

Postharvest senescence of fruit is a complex physiological and biochemical process. Fruit senescence is attributable to dissonance and accumulation of ROS. During the postharvest storage of the fruit, the function of the ROS scavenging system weakens. A large amount of ROS is produced inside the fruit, which leads to oxidative damage and tissue senescence. Literature shows that melatonin treatment effectively delayed the postharvest senescence and improved the quality of cassava (Hu et al., 2016; Ma et al., 2016), strawberry (Liu et al., 2018), jujubes (Tang et al., 2020), pepper (Korkmaz et al., 2021), peach (Gao et al., 2016), and ginger (Ke et al., 2021). Zhang et al. (2018) also showed that the melatonin treatment markedly reduced the cell membrane permeability and suppressed the production of ROS and MDA in the postharvest litchi. The results also indicated that the melatonin treatment delayed the fruit senescence by reducing the membrane lipid peroxidation. In our experiments, the changes in the physiological characteristics and ROS-related metabolism of the melatonin-treated litchi samples are consistent with the findings reported in the literature. Moreover, Sun et al. (2020) confirmed that the destruction of cell membrane structure was one of the reasons for the browning of the harvested crops.

Among the ROS, $O_{2}^{-}$ and H_2_O_2_ are the reactive oxygen free radicals determined in this study. Although the ROS generated in plant can regulate the growth of the plants, excessive accumulation of these free radicals may lead to membrane lipid peroxidation and protein oxidation. The lipid and protein oxidation processes are the causes of fruit membrane damage (Chomkitichai et al., 2014; Zhou et al., 2016; Wang et al., 2018). A previous study has shown that melatonin effectively increased antioxidant enzyme activities (Malik et al., 2021). It also reduced cell membrane damage and MDA content. In this study, the melatonin treatment effectively decreased the $O_{2}^{-}$ production rate and amount of H_2_O_2_. Hence, it effectively reduced the carbonyl-forming rate of the fruit samples and delayed the membrane lipid peroxidation. Besides inhibiting ROS production, the treatment also eliminated ROS *via* the antioxidant system. Lipid peroxidation leads to a loss of cell membrane function and increases protein carbonyl content in fruit. The increase in protein carbonyl content of the fruit indicates an increasing degree of protein oxidation. Protein carbonylation in fruit peel is one of the markers showing fruit senescence (Ciacka et al., 2020). During protein oxidation, carbonylated protein is formed. Protein carbonylation in fruit causes browning of the fruit pericarp (Zuo et al., 2021). It is a non-enzymatic browning of fruit.

Polyphenols, flavonoids, and anthocyanins are important non-enzymatic antioxidants and free radical scavenging compounds in plants. Polyphenols are phytochemicals involved in the browning reactions, and total polyphenols content is a good indicator for determining the degree of oxidation and browning (Teng et al., 2020). Polyphenols remove the accumulated and excessive ROS and delay browning in a plant (Shekari et al., 2021). This study shows that prolonged storage of litchi caused degradation of anthocyanins as these compounds are the principal polyphenols in litchi peel. Anthocyanins have been reported to scavenge free radicals *via* electron transfer reaction pathway, and the mechanism involves regulation of antioxidant enzyme such as SOD (Khoo et al., 2017). Bioactive compounds containing catechol group have higher antioxidant activities (Li et al., 2022). These phytochemicals found in litchi peels help to retard the fruit senescence besides the exogenous melatonin. The result also shows that the melatonin-treated litchi samples had better fruit quality than the non-treated fruit stored at 4°C for up to 12 days. According to the latest research on melatonin, the melatonin treatment increased accumulation of total phenolics and flavonoids in Chinese dates, reduced oxidative stress, and maintained the fruit quality (Wang et al., 2021). These findings supported the results obtained from this study that the melatonin treatment increased amounts of phenolic antioxidants in the litchi samples. Thus it reduced the browning rate and maintained the fruit quality. Anthocyanins in the litchi peel can also help scavenge free radicals and keep the freshness of the fruit besides its color to attract pollinators.

On the other hand, the antioxidant-related enzymes, such as SOD, CAT, APX, and GR, constitute an enzymatic antioxidant system in plants. Melatonin has been shown to effectively increase the activities of enzymes related to the oxidative defenses in apple (Sun et al., 2021a), maize (Malik et al., 2021), strawberry (Wu et al., 2021), and soybean (Zhang et al., 2020). The exogenous melatonin treatment also increased the endogenous melatonin accumulation in the litchi samples during storage (Hu et al., 2018; Ze et al., 2021). In this study, the high endogenous melatonin in the litchi samples could be attributed to the melatonin treatment. The accumulation of endogenous melatonin in melatonin-treated tomatoes could be triggered by the defense response to low-temperature stress (Sharafi et al., 2019). Wang et al. (2019) also speculated that a high endogenous melatonin level was one of the reasons for the delay in the senescence of sweet cherries. Therefore, endogenous melatonin plays a role in activating antioxidant defense mechanism to regulate the response of litchi to low-temperature stress besides helping to reverse the reactive oxygen-related protein damage in litchi (Liu et al., 2021a).

Some bio-oxidase and antioxidant enzyme activities depend on the gene expressions in antioxidant-related enzymes (Lee et al., 2007). In this study, we selectively determined the gene expressions in a few biological oxidation-related enzymes such as SOD and PPO. They are antioxidant protective and oxidative enzymes. The melatonin treatment upregulated the Fe-SOD gene expression. It also contributed to the defense against ROS. Moreover, the PPO gene downregulation was observed for the melatonin-treated litchi samples. On the contrary, several studies have shown that the Msr-related genes regulated oxidative stress by mediating sulfoxylation of methionine, participated in the redox regulation in the organisms (Jiang et al., 2017, 2018; Moskovitz and Smith, 2021), and also repaired oxidatively damaged protein (Aussel and Ezraty, 2021; Xiao et al., 2021b).

A previous study showed that the oxidized Met110 and Met125 residues of calmodulin (CaM) in the litchi peel could be repaired by the upregulation of LcMsrA1 and LcMsrB1 genes (Jiang et al., 2018). Another study reported that the expressions of Msr-related genes in the litchi samples were downregulated during prolonged storage at a room temperature of 25°C (Zhang et al., 2018). Jiang et al. (2017) also confirmed that the expressions of MsrA7 and CaM1 genes in the banana peel tissues could help to regulate antioxidant response. The gene expressions link to the regulation of banana fruit ripening and senescence. This study also indicated that the litchi sample treated with high-dose melatonin upregulated the LcMsrA1, LcMsrA2, and LcMsrB1 expressions. The relative expression of these genes might trigger the repair of damaged protein in the fruit peel, regulate ROS metabolism, and delay senescence or browning of the litchi peel.

Principal component analysis data revealed the physiological factors that affected the senescence of litchi during cold storage. These data showed that $O_{2}^{-}$ , MDA, and endogenous melatonin content were the indices of the main components. These components could distinguish the differences between the melatonin-treated litchi and control samples. Also, the mean comprehensive score of PCA showed that the melatonin treatment could maintain litchi quality. During the low-temperature storage, the overall scores of the melatonin-treated litchi samples were higher than that of the control sample. Therefore, postharvest quality of the melatonin-treated litchi was maintained if stored at 4°C for up to 9 days. These findings provide new knowledge on quality control and the potential application of melatonin in postharvest preservation of litchi.

## Conclusion

In this study, the melatonin treatment effectively maintained litchi freshness and its physicochemical properties for up to 9 days. The treatment inhibited ROS metabolism and increased the amounts of bioactive substances. It also enhanced the antioxidant enzyme activities and reduced the levels of enzymes related to peel browning. Melatonin treatment further increased the accumulation of endogenous melatonin in the litchi peel. The upregulated expressions of Msr-related genes could be attributed to the increased endogenous melatonin level in the fruit samples. The postharvest storage of litchi with the 0.6 mmol·L^−1^ melatonin treatment showed the best preservative effect. It regulated physiological and biochemical changes in fresh litchi to prolong its shelf life. Therefore, melatonin treatment is effective for postharvest preservation of litchi during cold storage.

## Data availability statement

The original contributions presented in the study are included in the article/Supplementary material, further inquiries can be directed to the corresponding authors.

## Author contributions

JX, ZQ, and XD conceptualized and designed the study. JX, ZQ, and JP collected the material, performed the experiments and wrote the manuscript. JX, ZQ, and HEK analyzed the data. JL, XL, HEK, and XD reviewed and edited the manuscript. All authors contributed to the article and approved the submitted version.

## Funding

This study was financially supported by the Guangxi Science and Technology Program (no. AD20297088) and the National Natural Science Foundation of China (grant no. 31760472).

## Conflict of interest

JX is an employee of the Changzhou Institute of Materia Medica Co., Ltd, Changzhou, China.

The remaining authors declare that the research was conducted in the absence of any commercial or financial relationships that could be construed as a potential conflict of interest.

## Publisher’s note

All claims expressed in this article are solely those of the authors and do not necessarily represent those of their affiliated organizations, or those of the publisher, the editors and the reviewers. Any product that may be evaluated in this article, or claim that may be made by its manufacturer, is not guaranteed or endorsed by the publisher.
