# Supplementary figures and images for: Melatonin treatment improves postharvest quality and regulates reactive oxygen species metabolism in “Feizixiao” litchi based on principal component analysis
Source: Front Plant Sci. 2022 Aug 11;13:965345. doi: 10.3389/fpls.2022.965345 (PMC9403734; doi:10.3389/fpls.2022.965345)

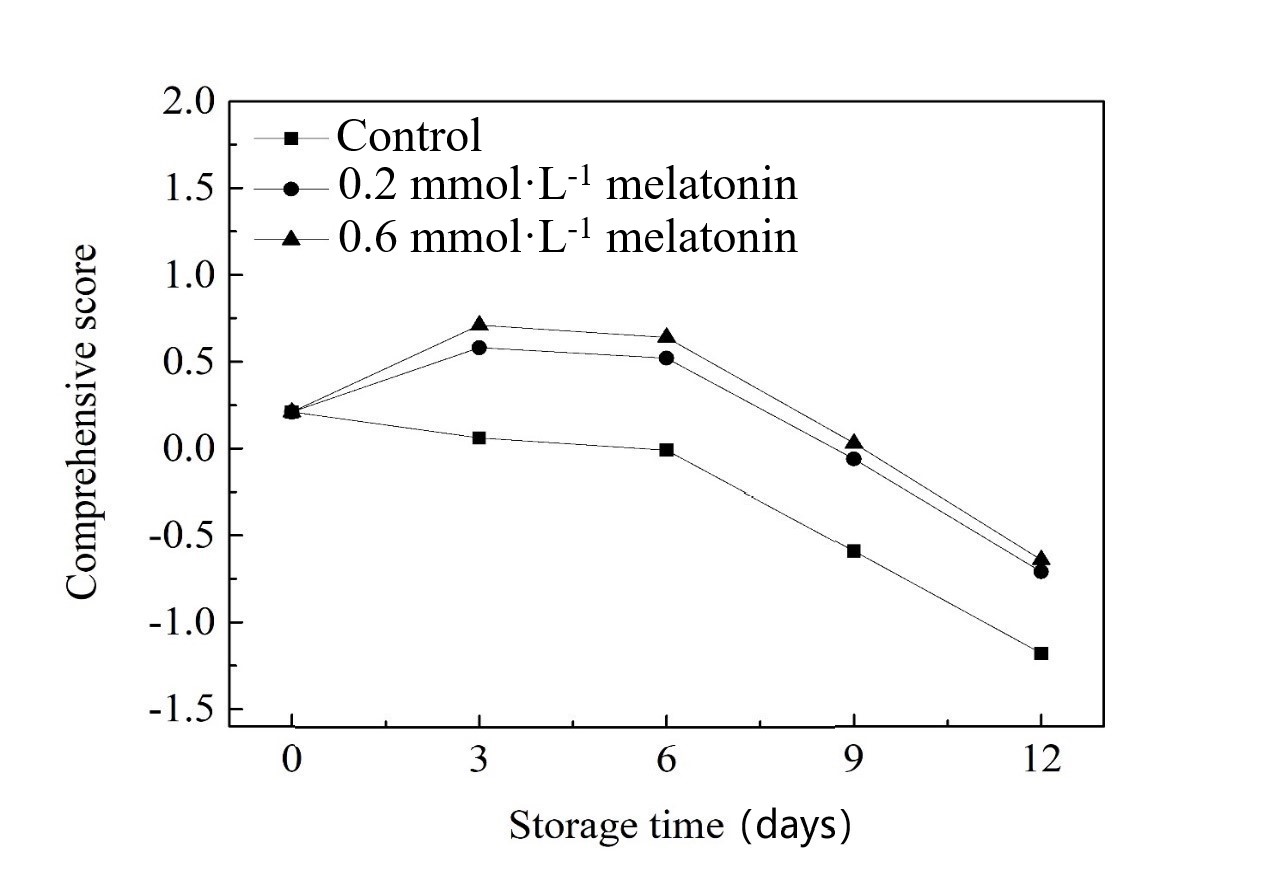

Supplement: Supplementary file 1 [file Image_1.JPEG]
